# Unraveling the Wide Spectrum of Melanoma Biomarkers

**DOI:** 10.3390/diagnostics11081341

**Published:** 2021-07-26

**Authors:** Antonios Revythis, Sidrah Shah, Mikolaj Kutka, Michele Moschetta, Mehmet Akif Ozturk, George Pappas-Gogos, Evangelia Ioannidou, Matin Sheriff, Elie Rassy, Stergios Boussios

**Affiliations:** 1 Department of Medical Oncology, Medway NHS Foundation Trust, Windmill Road, Gillingham ME7 5NY, UK; antonios.revythis@nhs.net (A.R.); sidrah.shah@nhs.net (S.S.); mikolaj.kutka@nhs.net (M.K.); 2 CHUV, Lausanne University Hospital, Rue du Bugnon, 21 CH-1011 Lausanne, Switzerland; michelemoschetta1@gmail.com; 3 Department of Internal Medicine, School of Medicine, Bahcesehir University, Istanbul 34353, Turkey; ozturkakif@yahoo.com; 4 Department of Surgery, University Hospital of Ioannina, 45111 Ioannina, Greece; pappasg8@gmail.com; 5 Department of Paediatrics and Child Health, West Suffolk Hospital NHS Foundation Trust, Hardwick Lane, Bury St Edmunds IP33 2QZ, UK; ioannidoueva@gmail.com; 6 Department of Urology, Medway NHS Foundation Trust, Windmill Road, Gillingham ME7 5NY, UK; matin.sheriff@nhs.net; 7 Department of Cancer Medicine, Gustave Roussy Institut, 94805 Villejuif, France; elie.rassy@hotmail.com; 8 Faculty of Life Sciences & Medicine, School of Cancer & Pharmaceutical Sciences, King’s College London, London SE1 9RT, UK; 9 AELIA Organization, 9th Km Thessaloniki-Thermi, 57001 Thessaloniki, Greece

**Keywords:** melanoma, biomarkers, molecular pathology, genetic mutations, prognosis

## Abstract

The use of biomarkers in medicine has become essential in clinical practice in order to help with diagnosis, prognostication and prediction of treatment response. Since Alexander Breslow’s original report on “melanoma and prognostic values of thickness”, providing the first biomarker for melanoma, many promising new biomarkers have followed. These include serum markers, such as lactate dehydrogenase and S100 calcium-binding protein B. However, as our understanding of the DNA mutational profile progresses, new gene targets and proteins have been identified. These include point mutations, such as mutations of the BRAF gene and tumour suppressor gene tP53. At present, only a small number of the available biomarkers are being utilised, but this may soon change as more studies are published. The aim of this article is to provide a comprehensive review of melanoma biomarkers and their utility for current and, potentially, future clinical practice.

## 1. Introduction

It is estimated that melanoma will be the 19th most common worldwide primary site of new cancers in both sexes in 2020, with 324,635 cases [1]. Very recently, immune evasion by cancer cells has become an important therapeutic target [2]. The prognosis varies according to the stage of the disease, from almost 99% 5-year survival rate in localised disease to approximately 27% when distant metastases are present [3].

As such, being able to predict which patients have the highest risk of developing distant metastases is quite important. Tumour biomarkers can be useful in predicting the risk of metastases and thus prognosis. Some of them can also have a diagnostic use. The use of serum biomarkers, such as lactate dehydrogenase (LDH) or S100b, is recommended in some guidelines, while the use of other serum biomarkers, such as melanoma inhibitory activity (MIA) and vascular endothelial growth factor (VEGF) is limited due to low specificity and limited clinical usability [4,5,6]. DNA point mutations in melanoma represent another important biomarker that can guide patient selection and predict treatment response, and are currently recommended by all clinical guidelines. For example, mutations of the Mitogen-Activated Protein kinase (MAPK) pathway, most commonly identified in *BRAF* and *NRAS*, have been correlated with shorter survival outcomes and response to selective inhibitors of BRAF mutant protein, such as dabrafenib and vemurafenib [7,8,9]. Other gene mutations offer similar correlations and represent potential therapeutic targets, such as *NRAS* gene mutations. The emergence of circulating tumour DNA (ctDNA) as an alternative source of DNA for genomic studies may become in the future the basis in clinical practice for tumor mutational analysis, staging of disease and consequent prognostication [10].

The aim of this review is to provide a comprehensive list of serum and DNA biomarkers currently under investigation in melanoma, and future potential applications.

## 2. Prognostic Tissue Biomarkers

In Alexander Breslow’s report in 1970, tumour thickness and cross-sectional tumour area were identified as prognostic variables reflecting tumour burden. The thickness of the primary tumour is considered a significant prognostic factor for stage I and II melanoma; overall, 5-year survival rates in stage III melanoma are based on thickness. When the thickness is less than 1 mm, the 5-year survival rate is 53%; while when it is 1–2 mm, 5-year survival rate is 47%; when 2–4 mm, 5-year survival rate is 40%; and when over 4 mm 5-year survival rate falls to 34% [11]. However, thickness of the primary tumour was not found to be prognostic after tumour metastasis (stage IV). Melanoma ulceration is another important prognostic factor. In literature, there have been two possible explanations of the adverse prognostic value of ulceration in primary melanoma. One possibility is that melanoma ulceration could directly enable dissemination of the tumour. Alternatively, it could be that ulceration is a biological attribute of tumours with a predisposition to disseminate.

Proliferative activity of the tumour and overexpression of *c-myc* have been found to favour both dissemination and ulceration of the primary melanoma [12,13,14]. The hypothesis that melanoma ulceration directly enables the dissemination of the tumour through alterations in the local environment has been indicated in studies on the interactions of melanocytes and keratinocytes [15,16]. These studies indicate that ulceration may provide melanoma cells with a very effective way to interrupt the keratinocyte-mediated control that prevents melanocyte transformation. Mitotic activity of the primary tumour has also been investigated as a prognostic factor. In DNA replication genes of two pathways are over-represented: replication origins firing (ROF) genes and the separation of sister-chromatids by securin. For example, overexpression of ROF genes in melanoma is associated with poor prognosis [17]. Expression of *MCM4* and *MCM6* genes is associated with metastasis-free survival and overall survival (OS) [17]. Securin is encoded by the *hPTTG* gene, which acts as an oncogene. Its expression is seen via immunohistochemical staining in the vertical growth phase but not in the radial growth phase of melanoma [18]. Some promising new prognostic tissue biomarkers have also been reported in the literature, including cycloxygenase 1–3 (COX1-3), galectin-3 molecule, matrix metalloproteinases (MMP), and chondroitin sulfate proteoglycan 4 (*CSPG4*). COX1-3 converts arachidonic acid to prostaglandin. In Becker et al., COX-2 staining intensity was found to correlate to Breslow thickness in melanoma [19]. Kuzbicki et al. also showed a higher COX-2 staining intensity in melanoma than in benign nevi [20]. The galectin-3 molecule is secreted by inflammatory cells, and has been associated with tumour progression and metastasis in melanoma [21]. Galectin 3 and tumour size were found to be inversely related and correlated with OS [22]. MMPs are key to remodeling of the tumour tissue microenvironment. MMP-1 and MMP-3 positive melanoma metastases were associated with reduced disease-free survival (DFS) [23]. CSPG4 is believed to be essential in cell adhesion, melanoma migration and metastasis [24]; over 80% of melanomas have been found to be expressing *CSPG4*. However, it can be found in any disease stage and there is no concrete evidence that it correlates to disease progression [25]. Finally, several recent studies have demonstrated that the receptor for advance glycation end products (RAGE) signaling from both melanoma and non-melanoma cells (fibroblasts, immune cells, endothelial cells) in the tumor microenvironment represents an important element in the process of melanoma tumor growth. The RAGE/ligand axis appears to support the association between chronic inflammation and immunosuppression. Therefore, targeting RAGE in melanoma tumors could be therapeutically beneficial [26].

## 3. Prognostic Serum Biomarkers

The use of serum biomarker assays may identify the presence of residual or recurrent disease prior to imaging studies and relevant radiological evidence. From the therapeutic perspective, this is important, as the prediction or early identification of distant metastasis would enable the timely initiation of systemic therapy in adjuvant or metastatic settings [27].

### 3.1. Lactate Dehydrogenase (LDH)

LDH catalyzes the conversion of pyruvate to lactate in hypoxic or anoxic conditions. An elevated level of LDH is believed to be due to spillage into the bloodstream when melanoma cells outgrow their blood supply [4]. High levels of LDH are associated with worse prognoses, independently of site or number of metastases [28]. In the American Joint Committee on Cancer (AJCC) melanoma staging system, patients with distant metastasis and elevated LDH levels are considered stage IV M1c [4]. Patients with stage IV disease and normal serum LDH at initial staging have 1-year OS of 65% and 2-year OS of 40%. With elevated LDH levels, 1-year and 2-year OS are 32 and 18%, respectively [4]. Apart from its prognostic value, in patients treated with a combination of dabrafenib and trametinib, LDH was shown to be associated with poorer outcomes [29]. Moreover, when LDH decreases by more than 27.3% from the baseline, this has been associated with radiological response to immunotherapy [30].

### 3.2. S100 β

S100 proteins are implicated in a multitude of cell functions. As early as the 1980s, S100 β was found to be expressed in human melanoma cell lines and was proposed as a marker that could aid in diagnosis of melanoma [31].

However, S100 β can be found in abnormal levels in many pathological conditions, including liver, brain and renal injury, inflammatory and infectious processes [32].

In 2008, Mocellin et al. published a meta-analysis of 22 series, with a total of 3393 patients with malignant melanoma at all stages. This revealed that positive serum 100B was associated with reduced survival (hazard ratio [HR]: 2.23; connfidence interval [CI]: 1.92–2.58; *p* < 0.0001) [33]. Abraha et al. displayed a correlation between serum S100B levels and Breslow thickness. Serum S100 β > 0.2 μg/L and primary melanoma tumour thickness > 4 mm combined had sensitivity of 91% and specificity of 95% as predictors for disseminated disease, and consequently may inform prognosis at the point of diagnosis [34]. However, this was not confirmed in other studies of multivariate analyses, where levels of serum S100 β did not show clinical prognostic value [35]. Overall, the evidence for routine use of serum S100 β as a prognostic marker in melanoma is limited. This is due to small sample sizes, lack of proven significance in multivariate studies and mismatch of disease stages across studies [33]. However, measurement of serum S100 β in patients with Breslow > 1 mm lesions is recommended every 3–6 months in German and Swiss guidelines [36,37].

### 3.3. Melanoma Inhibitory Activity (MIA)

Melanoma inhibitory activity (MIA) is secreted by melanoma cells and is a regulatory growth factor [38]. MIA was proposed as a melanoma biomarker, because it is not expressed in benign human melanocytes or benign melanocytic nevi, but is strongly expressed in malignant melanoma cells [39]. Higher levels of MIA were linked with more advanced stages of melanoma and worse prognosis [5]. This was first shown in a German study that included over 830 blood samples of 326 patients with malignant melanoma of all stages. The cutoff was set at 9.8 ng/mL. In stage I and II patients, elevated MIA concentrations were found in 5.6%. This increased to 60% in patients with stage III and 89.5% in stage IV melanoma. Patients at stage III or IV that underwent resection or treatment with irradiation or chemotherapy prior to the study had MIA levels below the cutoff. Notably, all patients with reduced MIA levels in all stages did not develop further metastases during the follow-up period. In patients displaying a significant increase in MIA levels, metastases were detected either at the point of analysis, or in the following 2–6 months [40]. A comparative study in 373 melanoma patients among MIA, S100B, LDH and albumin showed that MIA was not superior to the use of S100 β or LDH; specifically, S100 β had the higher sensitivity (0.86) in newly diagnosed metastatic melanoma while MIA had the second highest (0.80); LDH sensitivity was lower at 0.48 and albumin lowest at 0.15. However, MIA had the lowest specificity (0.62), whilst albumin had the highest (0.99) [6].

### 3.4. Vascular Endothelial Growth Factor (VEGF)

VEGF is elevated in patients with advanced-stage melanoma. This was associated with negative immune effects, such as impaired dendritic cell function [41,42]. It was also linked with both elevated and decreased T helper 2 (Th2) cytokines. These were found to result in suppression of effective antitumour immunity. VEGF inhibitors can lead to improved dendritic cell function and reverse Th2 dominance, leading to Th1 polarity. These changes should in theory enhance tumour rejection [43]. Ugurel et al., in a study including 125 patients with stages I-IV melanoma, concluded that VEGF was found to be an independent prognostic marker for OS [44]. However, VEGF has not been found to be effective as a marker of disease progression. This was replicated in a study in 2005, although healthy individuals were found to have higher VEGF levels [45]. When used to monitor patients, VEGF potentially has high negative predictive value (90%) with low sensitivity, specificity and positive predictive value of 57.1%, 78% and 34.5% respectively.

### 3.5. Other Serum Biomarkers

Apart from those already discussed, there is a multitude of other promising serum biomarkers. Tumor associated antigen 90 immune complex (TA90IC) and its utility in melanoma has been indicated in several studies. In a comparative study between TA90IC, MIA and S100 β in stage III melanoma patients undergoing adjuvant immunotherapy, TA90IC was the earliest elevated marker and an independent predictor for survival and recurrence of melanoma [15]. Further studies support this, indicating that antiTA90 IgM can be an independent prognostic factor for melanoma [46]. The expression of TA90IC can be elevated in inflammatory processes, such as hepatitis with liver cirrhosis [15]. Tyrosinase is a marker specific for melanocytes and Schwann cells, which are normally not found in peripheral blood [47]. Several studies have been performed, with conflicting results. Some have shown that the presence of microRNAs (miRNA) in tyrosinase correlates with melanoma relapse progression [48,49]. However, variable levels of miRNA in stage III and IV melanoma indicate that blood tyrosinase level is not a dependable marker in metastatic disease [50,51]. Osteopontin’s role in cell death, tumour cell growth and recruitment of tumor promoting stromal cell has also been described [52,53,54]. In Maier et al., it was shown that a combination of S100 β and osteopontin may correlate with disease relapse and help identify patients at high risk of metastasis [55]. However, osteopontin can also be elevated in several autoimmune conditions. Interleukin-8 is a chemokine associated with inflammatory processes; it has been shown to promote angiogenesis and correlate with disease stage, survival, tumour burden and response to treatment [56]. Melanoma Antigen Gene A3 protein (MAGE-A3) is part of a family of proteins whose genes are normally silent, except in male germline cells. This is not the case in melanoma and other tumours though [57]; indeed, elevated levels are found in early melanoma stages. Nevertheless, its use as a prognostic factor has yet to be proved [58]. YKL-40 is a glycoprotein secreted by activated neutrophils and macrophages. Elevated levels can be seen in non-malignant diseases, but were also found to be an independent prognostic biomarker for poor survival in breast, lung, colon, ovary and kidney cancers [59]. Their use alone or in conjunction with LDH as an independent prognostic marker was shown by Schmidt et al. [60]. In the same study, serum level of YKL-40 at diagnosis was found to be an independent factor for survival [61]. However, YKL-40 has yet to receive approval by the Food and Drug Administration (FDA) to be used as a biomarker in the United States [62]. In addition, medications such as IL-2 and IFN-a2b increase YKL-40 expression and can cause false negative results [63]. Cytoplasmic melanoma-associated antigen (CYT-MAA) is produced in normal and tumour cells alike. However, its levels are elevated in melanoma. Although it is not sensitive or specific, it has been linked with disease recurrence and progression, as well as potentially with response to immunotherapy [64]. Melanotransferrin (MTF) is expressed in normal adult, fetal and tumour cells [65]. It is commonly found in exocrine tissues, such as salivary glands and the pancreas, as well as the epididymis [66]. Its exact role in melanoma is not yet known. However, it is thought to contribute to angiogenesis, tumour proliferation and tumour genesis [66,67]. Microphthalmia-associated transcription factor (MITF) contributes to the regulation of melanocytes’ development, differentiation and function [68]. MITF is sensitive and specific in identifying melanoma cells [69]. Its levels are inversely proportional to melanoma cell invasiveness [70]. The identification of MITF after treatment indicates metastatic disease and worse outcomes in melanoma patients [71]. Glycoprotein 100 (gp100) is normally expressed in adult melanocytes. However, levels are increased in neonatal cells and melanoma, albeit those levels vary [72]. Despite this, gp100 is not specific and was not proven to correlate with response to treatment [73,74]. Lastly, elevated C reactive protein (CRP) and interleukin 6 (IL-6) levels were found to be linked to reduced survival and treatment resistance [74,75]. Despite this, CRP was found to be an independent predictor for survival [76].

Interestingly enough, protein levels can vary significantly between serum and plasma, due to the storage conditions, the method of blood fractionation and the properties of the specific analysed proteins. As such, there are significant discrepancies in the literature regarding protein levels in plasma and serum; nevertheless, it has been shown that plasma has better reproducibility in protein measurement. Table 1 summarises the serum biomarkers that were described here. Among them, at least S100 β, MIA, VEGF, osteopontin, and interleukin 8 (IL-8) are relevant in plasma [77].

## 4. DNA Markers

Epigenetic alterations: hypermethylation.

Epigenetics is the study of potentially inheritable changes in the phenotype that do not involve alterations in the DNA sequence [78]. Epigenetic DNA changes are crucial in determining which genes are silenced or expressed in processes like cell differentiation, cell growth and immune response, and have been extensively investigated in melanoma [79]. DNA methylation occurs in 5-cytosine exclusively to produce methylcytosine, and the majority of this is seen in CpG dinucleotides [80]. There are large clusters of CpG dinucleotides across all of the genome, referred to as CpG islands, typically in promoter regions [78]. Hypermethylation of these promoter regions in cancer-related genes can facilitate tumour progression [81]. This is because DNA promoter methylation can inactivate tumour suppressor genes (TSGs) [82]. Methylation changes in the CpG island promoter regions in TSGs or other tumour-related genes have been observed in malignant cutaneous melanoma [83]. This phenomenon in melanoma has been named the CpG island methylator phenotype (CIMP) [84].

The Ras association domain family 1 A (*RASSF1A*) is a TSG that can control the cell cycle, promote apoptosis and maintain the genome [85,86]. In breast, lung and liver cancer cells, RASSF1A expression was lower; studies have demonstrated that this lower expression is due to methylation of its promoter region [82]. Spugnardi et al. demonstrated that 55% of malignant melanoma tumours had a hypermethylated *RASSF1A* gene, which is one of the most significant epigenetic alterations and loss of TSGs reported in cutaneous melanoma [87]. Unlike other cancers, such as bladder cancer, it is undetermined whether RASSF1A hypermethylation is related to poorer disease outcome and survival in melanoma [85]. Tanemura et al. determined that *RASSF1A* methylation was present in nearly 50% of stage IV melanoma compared to no methylation in stages I and II; this indicates that RASSF1A could signify progression and prognosis in melanoma, but further studies must explore this further [83].

Hoon et al. further explored DNA methylation of CpG islands in the promoter regions of TSGs [88]. TSGs were assessed using methylation-specific polymerase chain reaction (MSP) in 130 cutaneous tumours and 15 melanoma cell lines. They found four TSGs were especially hypermethylated in 86 metastatic melanoma tumour specimens: *RASSF1A* (57%), retinoic acid receptor responder protein 2 (*RARRES2*) (70%), death-associated protein kinase (DAPK) (19%) and O^6^-methylguanine DNA methyltransferase (*MGMT*) (34%). Hypermethylation in *RARRES2* was 70% in both primary and metastatic melanoma, but *RASSF1A*, *DAPK* and *MGMT* had significantly lower hypermethylation in primary melanoma versus metastatic melanoma. Similarly, the most hypermethylated gene in melanoma cell lines was *RASSF1A* (80%), followed by *RARRES2* (53%) and *MGMT* (27%). Overall, hypermethylation was higher in metastatic melanoma compared to primary melanoma, indicating a possible role in tumour progression, but TSG hypermethylation was not significantly associated with disease outcome or OS. However, RARRES2 did correlate with Breslow thickness of the tumour (*p* = 0.009). This significant correlation has major implications, as Breslow thickness is a prognostic factor in localised and early-stage melanoma.

Another study analysed hypermethylation of *RASSF1A* and *RARRES2* in 37 melanoma patients with clinically positive lymph nodes [89]. Hypermethylation was observed in 16% of subjects in *RASSF1A* alone, 28% in *RARRES2* alone, and 14% in both. Furthermore, hypermethylation of *RARRES2* correlated with reduced DFS and OS. Other studies also observe that hypermethylation increases as melanoma progresses in its stages [90,91]. This suggests that increasing degrees of hypermethylation could be used to predict prognosis of melanoma in the future. As further TSGs involved in melanoma are identified, MSP will play a significant role in determining whether this hypermethylation influences melanoma progression and later metastasis [92]. More research is warranted to determine the accuracy of hypermethylation as a marker of prognosis.

### 4.1. Mutations of the Mitogen-Activated Protein Kinase (MAPK) Pathway Mutations

#### 4.1.1. BRAF Mutations

A whole-genome sequencing study in 2013 analysed the frequency of mutations across multiple cancers and identified melanoma as the most frequently mutated tumour [93]. The majority of melanomas have mutations in the mitogen-activated protein kinase (MAPK) pathway, a pathway vital for cell growth, proliferation and survival [7]. Mutations occurring in this pathway leads to dysregulated cell growth and cell cycle activation [94]. Oncogenic activation of the MAPK pathway mainly involves two mutations: *BRAF,* the most commonly mutated gene in 40–60% of cases, followed by *NRAS* in 15–30% of cases [95,96,97]. In melanoma, *BRAF* mutation occurs in 45–50% causes, specifically through mutations at the V600 codon [98]. The most common BRAF mutation is V600E, accounting for 80% of mutations in the gene, followed by V600K and V600R [95]. The V600E mutation is found in younger patients with superficial spreading melanoma or in areas not exposed to chronic sun-induced damage [8,95]. In contrast, the V600K mutation is found in older patients with melanoma due to chronic sun-induced damage, especially to areas such as the head and neck [99].

*BRAF* mutations may be correlated with ultraviolet (UV) exposure [95]. Whole-genome sequencing by Colebatch et al. found that mutational load of BRAF was positively correlated with UV exposure in benign melanocytic naevi [99]. Similarly, Bauer et al. found there were no *BRAF* mutations in congenital melanocytic naevi, further emphasising that UV exposure could induce these mutations in the skin [100]. Further research is needed to identify if this mutation could be useful in tracking the transformation of benign naevi to malignant melanoma. *BRAF* mutations also have implications for prognosis. Recent research has established that *BRAF* mutations could be linked with shorter DFS in early stages of melanoma [95]. Furthermore, BRAF-mutated melanoma is connected with shorter survival in stage IV disease and V600E expressed in the nucleus (rather than the cytoplasm) is associated with more advanced tumour staging, lymph node metastasis and depth of invasion [92,101]. With the identification of *BRAF* and its significant role in melanoma, therapies have been developed to target its specific inhibition.

Selective inhibitors of V600-mutated kinase, dabrafenib and vemurafenib, have been associated with improved OS and DFS [102]. A phase three randomised clinical trial compared vemurafenib with chemotherapy dacarbazine in 675 previously untreated subjects having metastatic melanoma with the *BRAF* V600E mutation [9]. At six months, OS was 84% in the vemurafenib group compared to 64% in the dacarbazine group. Furthermore, vemurafenib had a relative reduction of 74% in risk of disease progression and death compared to dacarbazine (*p* < 0.001). Although dabrafenib and vemurafenib are both promising and beneficial treatments in metastatic melanoma, their clinical responses differ according to the type of mutation present (V600K melanomas with brain metastases had a lower response rate of 7% to dabrafenib treatment compared to 39% with *BRAF*-V600E melanomas [101,103,104]). Further research is needed to investigate response rates in different mutations as well as in earlier stages of melanoma; these studies could then be used to predict prognosis with the given treatments [101].

However, the most concerning challenge currently is resistance to these BRAF inhibitors due to increasing new mutations and upregulation of receptor tyrosine kinase (RTK) or *NRAS* [92,105]. Consequently, combination therapies using BRAF and MEK inhibitors such as cobimetinib and trametinib are becoming the standard of melanoma treatment [106,107]. The COMBI-d and COMBI-v double-blind phase three trials showed combination therapy with dabrafenib and trametinib had higher DFS and OS when compared to monotherapy of either dabrafenib or vemurafenib [11,108]. Furthermore, combination therapy with dabrafenib and trametinib was seen to reduce cutaneous squamous-cell carcinoma and keratoacanthoma, occurring in 1% of patients compared to 18% receiving vemurafenib alone [104]. Encorafenib is a BRAF inhibitor which has a 10-fold longer half-life than vemurafenib or dabrafenib [109]. It is being combined with the MEK inhibitor binimetinib to demonstrate more potent inhibition and efficacy in BRAF-mutated melanomas [109]. Further treatments to resolve resistance are being investigated, which include the addition of MAPK, CDK, Rho kinase or immune checkpoint inhibitors.

#### 4.1.2. NRAS Mutations

*NRAS* is a GTPase protein that integrates signals from multiple RTKs [110]. *NRAS* mutations activate MAPK signaling similarly to *BRAF* mutations; these activated signalling pathways lead to dysregulated cell cycles along with cell proliferation and further survival pathways [111].

*NRAS* mutations have been observed in 15–25% of melanomas and typically develop later in life after UV exposure and on the peripheral extremities [104,112]. *NRAS* mutations usually occur independently of *BRAF* mutations, but 10–20% of melanomas have point mutations in *NRAS* codons 12, 13 or 61 that may be mutually exclusive with *BRAF* mutations [113,114,115]. If occurring independently, *NRAS* mutations may be able to bypass *BRAF* and signal through *CRAF* instead [116]. *NRAS* mutations have been observed in melanoma, which suggests that UV radiation may play a significant role in introducing these mutations [117]. However, these mutations may also arise independently of UV radiation as they have been found in congenital melanocytic naevi as well; in these cases, detecting the signature UV radiations may help to diagnose the melanoma [96,118]. *NRAS* mutations are also found in melanocytic and dysplastic naevi and melanomas with a high mutation load [102,119].

It is unclear if NRAS has any prognostic value if identified, but *NRAS* expression is associated with higher tumour staging and lower grades of tumour infiltrating lymphocytes [120]. In comparison with wild-type melanoma, *NRAS* mutations may result in a significantly worse melanoma-specific survival rate [121]. This may be explained histologically by their inducement of thicker lesions, higher mitotic activity and increased lymph node metastasis [122]. *NRAS* mutations activate the RAF/MEK/MAPK signalling pathway in a similar fashion to *BRAF* [123]. There has been a focus on inhibiting downstream components of the Ras signaling pathway, in particular the farnesyl transferase inhibitors (FTIs) [104,124,125,126]. The toxicity observed is attributed to the fact that FTIs inhibit other proteins that require farnesylation [127].

#### 4.1.3. GNAQ/GNA11 Mutations

*GNAQ* and *GNA11* genes code for G alpha subunits of G proteins that act with G-protein coupled receptors [128]. The conversion of GDP to GTP allows G protein and G-protein coupled receptor signaling and subsequent activation of G proteins; for G proteins to become inactive, GTPase hydrolyses GTP to GDP [129]. These genes cause GTP to be constantly bound to the G protein and result in downstream signaling [130,131,132,133].

Identifying *GNAQ* or *GNA11* mutations can be useful to diagnose uveal melanoma and differentiate it from other types of melanomas and melanoma of undefined origin [134]. While they can be present in cutaneous melanoma, these cases are rare [130,135]. In contrast to their diagnostic value, their use as a prognostic marker has limited evidence. Sheng et al. found the median OS to be shorter for patients with *GNAQ* and *GNA11* mutated mucosal melanoma compared to the wild-type subsets [136]. In contrast, other studies have shown *GNAQ* and *GNA11* mutations are not associated with poor patient outcomes or disease progression in uveal melanoma, which could be due to the mutations being initial steps in the development of melanoma [137,138]. Furthermore, there is no significant difference in OS or DFS in patients holding *GNAQ* mutations compared to *GNA11* mutated melanomas [136].

### 4.2. Neurofibromatosis 1 (NF1)

*NF1* gene encodes a protein (neurofibromin) which acts as a negative regulator of the RAS-dependent pathway. It is known to cause tumours with mostly neuroectodermal origin that consequently can often be found in melanoma [139]. Desmoplastic melanomas have fewer DNA copy number alterations than other melanoma subtypes; nevertheless, the few focal deletions that have been observed targeting *CDKN2A* and *NF1* [140]. *NF1* mutations are found in up to 45–93% of these melanomas [141,142,143].

In an analysis of 1058 patients’ pathological reports with advanced melanoma treated with anti-PD-1 or anti-PD-L1 antibodies, Eroglu et al. identified 60 patients with advanced desmoplastic melanomas, who overall had a high response rate to PD-1 blockade. Whole-exome sequencing of 17 of these patients revealed that 14 had *NF1* mutations (82.4%) [144]. These findings suggest that, despite the dense fibrous stroma that had been expected to limit PD-1/PD-L1 effects, the blockade may be effective in patients with desmoplastic melanomas with *NF1* mutation. *NF1* mutation can develop simultaneously with *BRAF* mutation, and as a result melanomagenesis is enhanced and resistance to vemurafenib increased [140]. At the same time, preliminary data support the possibility of increased sensitivity to MEK inhibition with trametinib [145].

Finally, there is a subset of patients who do not express BRAF, NRAS, or NF1 mutations. Most often these tumors may have other MAPK mutations, AKT3 overexpression, or changes in cell cycle pathways and most likely need a novel therapeutic approach.

### 4.3. PI3K/AKT/mTOR Pathway Mutations

PI3K/AKT/mTOR is a critical regulator of many physiological processes and essential to the aggressive nature of the tumour, as this pathway promotes cellular growth and survival [146].

AKT family member mutations are often dysregulated in melanoma and have been identified in up to 43–60% of melanoma cases [147]. *PTEN,* which classily dampens the PI3K/AKT/mTOR growth-promoting signaling cascade, is noted in 38% of patients with primary melanoma and 58% of patients with metastatic disease [148]. Changes in *PTEN* and *BRAF* pathway often co-exist, theoretically allowing dysregulation of both the MAPK and PI3K pathways at the same time [146]. Hence, it might be possible that PI3K inhibitors may afford some benefit to patients with *PTEN* and/or AKT-mutant melanomas. Rapamycin and its analogues were among the first to be tried, inhibiting mTOR. One of the reasons is that they are known to be well-tolerated clinically, as demonstrated by long-standing use in patients who have undergone organ transplantation. However, mTOR inhibitors have not demonstrated significant clinical activity as single agents in metastatic melanoma patients, nor when combined with RAF inhibitors [149]. One of the potential reasons is the complexities of pathway inhibition in systems with significant cross-talk.

### 4.4. KIT Mutations

*c-KIT* is a transmembrane receptor tyrosine kinase. When binding a stem cell factor, it results in activation of several signaling pathways, thereby mediating cancer cell growth, proliferation, invasion, metastasis, and inhibition of apoptosis [150]. The majority of *c-KIT* mutations are found in mucosal and acral melanomas, as well as in melanomas arising from skin [151]. Past genotyping has shown that they are almost always mutually exclusive with *BRAF* and *NRAS* mutations [96]. Expression of *KIT* is not uniform across the tumor; the highest levels of *KIT* expression are seen at the leading edge of tissue invasion, indicating a key role it may have in promoting metastasis [151]. The presence of *c-KIT* mutations has shown to be associated with worse survival as compared with wild-type melanomas.

Unfortunately, because of the relative rarity of *c-KIT* mutations (1–7%), the availability of targeted therapy to treat this type of melanoma is limited. However, responses to tyrosine kinase inhibitors (imatinib, sorafenib) have been reported in patients with *KIT*-mutant melanoma [152,153].

### 4.5. CDKN2A Mutations

*CDKN2A* gene encodes the *p16* protein, thus mutations in this gene result in hyperphosphorylation of retinoblastoma protein. Hence, it regulates intracellular oxidative stress in a cell cycle-independent manner [154].

Goldstein et al. found that mutations in the *CDKN2A* gene are the most common alteration in hereditary melanoma. These mutations are found in 40% of families with high incidence of melanomas [155]. Moreover, based on pathological analysis of *CDKN2A*-mutated familial melanomas, it was found to be strongly associated with the more aggressive superficial spreading subtype [156]. Furthermore, *CDKN2A* germline families have a higher inherited risk of developing pancreatic cancer [157].

In terms of prognostic value, a Swedish study demonstrated that the *CDKN2A* mutation was associated with a younger age at onset and worse survival, whereas an Italian cohort study did not correlate the mutation with worse survival [158,159].

### 4.6. BAP1 Mutations

BAP1 is a tumor suppressor gene and is often associated with metastatic uveal melanoma [132]. There is a known correlation with tumor aggression and worse prognosis in uveal melanoma, as well as greater risk of metastasis [160]. *BAP1* tumor predisposition syndrome (BAP1-TPDS) is a cancer syndrome that apart from the aforementioned uveal melanoma predisposes patients to other malignant disorders, including renal cell and basal cell carcinomas, lung and breast/ovarian cancers, meningioma, and malignant mesothelioma [160,161].

Therapeutic targeting of *BAP1* mutation poses a challenge, as its mechanism in melanoma development is still poorly understood. Not only is the identification of the function of *BAP1* responsible for its anticancer role unclear, but also the goal of therapy is complex as it aims to restore one or more functions of *BAP1*. Some studies are targeting alternative mechanisms of DNA repair. One focuses on poly (ADP-ribose) polymerase (PARP) emerging as a potential target for treatment. The main reason is its role in base-excision and nucleotide excision repair [162]. Therapeutic target of *BAP1* has focused on its role in DNA double-strand break repair via homologous recombination [163,164]. Indeed, there is an ongoing clinical trial of the PARP inhibitor niraparib in BAP1-deficient neoplasms including uveal melanoma (NCT03207347) [165].

### 4.7. The Role of Gene Fusions in Melanoma

Advances in next generation sequencing (NGS) have led to the identification of many important kinase fusions as the primary drive in melanoma, which may represent critical targets for molecular therapy [166].

*NTRK1* fusions typically arise from small deletions, whereas the *AGK-BRAF* fusion arises through an inversion. Due to the fact that most studies are RNA-based, many of the precise genomic mechanisms are not yet characterised. Most fusions have been identified with a variety of N-terminal partners. Kinases activated by these gene fusions include *ALK*, *RET*, *ROS1*, *NTRK1*, *NTRK3*, *MET*, *MAP3K8*, *MAP3K3*, *BRAF*, and *PRKCA*. The subsequent activation of downstream RAF/MEK/MAPK, PI3K/AKT/mTOR, and PLC pathways promotes cellular proliferation and migration. These fusions are mutually exclusive of one another, as well as of other driver mutations previously reported, such as *NRAS*, *HRAS*, *GNAQ*, *GNA11*, *NF1*, and *KIT*.

Fusions may be the initiating genomic event in 8–20% of melanomas [167]. From a therapeutic perspective, BRAF or MEK inhibitors may be effective, as they reduce tumor size and proliferation [168,169]. It seems that NGS has become a useful tool in screening for targetable fusions in advanced melanomas that lack characteristic driver mutations.

## 5. Molecular Profiling for Liquid Biomarker Discovery in Melanoma

### 5.1. Circulating Tumour DNA (ctDNA)

Circulating tumour DNA (ctDNA) is highly fragmented single or double-stranded DNA that is shed by tumour cells into the circulation [170,171]. ctDNA assays constitute a powerful tool for study of the molecular heterogeneity and clonal divergence of a malignancy. Levels of ctDNA can vary depending on tumour vascularity, location and cellular turnover [172,173]. Generally, undetectable ctDNA and favourable molecular profile carry a better response, progression-free survival and OS compared with detectable ctDNA at baseline or during treatment [10]. ctDNA may also be useful as a biomarker of disease recurrence after melanoma resection [174]. As expected, ctDNA is usually undetectable in early-stage melanomas [175]. In later stages, the presence of *BRAF* and *NRAS* mutations is associated with response to immunotherapy. Patients with an undetectable ctDNA either at baseline or during treatment achieve a better objective response as compared to those with detectable ctDNA at baseline which remained detectable during therapy [10]. Moreover, baseline levels of ctDNA were lower in melanoma patients who responded well to targeted therapy [176,177]. The analysis of methylated ctDNA using methylation-specific PCR in metastatic melanoma has yielded promising associations. Indeed, methylated RASSF1 may serve as an indicator of response to hormonal treatment [178]. ctDNA may be detected as loss of heterozygosity (LOH) in DNA microsatellites. This was seen in a study where the plasma of 76 patients with stage I to IV melanoma was analysed for microsatellite loss using 10 markers [179]. LOH of at least one marker was found in 50% of patients and was correlated with the stage of the disease. Taback et al. analysed preoperative and postoperative serum for LOH in 57 patients with melanoma of all stages. All of them were deemed surgically disease free [180]. Using a different set of markers, investigators found that LOH of at least one marker was found in 56% of patients and correlated to the disease stage. Fujimoto et al. attempted to detect LOH in a panel of four biomarkers and identify a correlation to response to biochemotherapy. Their study recruited 49 patients with stage IV melanoma and concluded that patients who did not respond to biochemotherapy had significantly more LOH [181]. The presence of LOH of 12q was also linked to significantly worse OS. Overall, the benefit of using detection of ctDNA mostly relates to easier sampling allowing multiple serial analysis, compared to sampling of the primary or metastatic melanoma via tumor biopsies.

### 5.2. MicroRNAs (miRNAs) and Long Noncoding RNAs (IncRNAs)

miRNAs are short noncoding RNA molecules (20–200 nucleotides) that regulate gene transcription processes, which in turn affect cell proliferation, apoptosis, cell differentiation and cell survival. Long noncoding RNAs (lncRNAs), with more than 200 nucleotides, also regulate transcriptional, post-transcriptional and epigenetic gene expression modulation [182]. In contrast to ctDNA, miRNAs and IncRNAs are relatively stable, because they are predominantly secreted in vesicles, or in complex with other proteins [182,183,184,185,186].

Identification of miRNAs and lncRNAs can provide valuable information in diagnosis and prognosis and offers predictive value in melanoma [187]. It seems that their use is limited due to low specificity and difficulty in attributing whether an increase in levels is due to cancer or due to other conditions, such as inflammation [188]. However, in the era of personalised medicine, the relationship between aberrant miRNA profile and response to therapeutic regimens should be further evaluated. Therapeutic targeting of miRNAs can impact the natural history of melanoma by enhancing sensitivity to both standard therapies and immune checkpoint inhibitors [189]. In particular, elevated levels of miRNA-221 have been identified in early melanomas, compared to healthy individuals. Increased levels were also linked to increased stage of disease [190]. In a recently published study, circulating miRNA-615–3p levels were consistently more efficient in detecting melanoma patients who developed progressive disease whilst treated with immune checkpoint inhibitors, as compared to LDH levels [191]. A panel of five miRNAs was used to classify primary melanoma patients as low or high risk of recurrence. In serial testing, dynamic changes reflected tumour burden [192]. Finally, a study demonstrated that specific circulating miRNA signatures may distinguish melanoma brain metastasis from other types of brain cancer metastases as well as primary glioblastomas [193]. Several lncRNAs were also found at high levels, including *SPRY4-IT1*, *BANCR*, *HOTAIR*, *UCA1* and *MALAT-1* [194]. In particular, levels of *UCA1* and *MALAT-1* were significantly higher in patients with melanoma compared to controls and were correlated to the stage of the disease [195]. Finally, the potential of targeting ncRNAs for the development of novel therapeutic strategies or for the optimization of the efficacy of standard treatments has been assessed in several studies [196].

DNA markers and molecular liquid biomarkers are listed in Table 2.

## 6. Predictive Markers of Response to Immunotherapy

The establishment of predictive biomarkers for checkpoint immunotherapy is hugely important in the era of personalised medicine.

Direct assessment of PD-L1 expression on tumour cells is a predictive biomarker of treatment response to anti-PD-1 or anti-PD-L1 therapies. Progression-free survival and OS are prolonged in PD-L1-positive versus PD-L1-negative patients [197]. However, even PD-L1-negative patients may obtain clinical benefit from anti-PD-1 or anti-PD-L1 therapies. Indeed, objective responses in PD-L1-negative patients—usually between 11–20%—have been reported in the literature, whilst melanoma patients reached an overall response of 41% with nivolumab monotherapy, and 54% with nivolumab plus ipilimumab in the CheckMate 067 study [197]. These data demonstrated that the negative predictive value of anti-PD-1 or anti-PD-L1 therapies is suboptimal (58 vs. 45% for nivolumab and nivolumab plus ipilimumab, respectively).

The use of mutational or neoantigen burdens has also been studied as a predictive biomarker in melanoma patients treated with immune checkpoint inhibitors. In a study of advanced melanoma patients who received CTLA-4 inhibitors (either ipilimumab or tremilimumab), a mutational load of more than 100 non-synonymous somatic mutations was associated with long-term clinical benefit [198]. This mutational load cutoff was characterised by longer OS compared with patients with a lower mutational load. A similar study of melanoma patients treated with ipilimumab demonstrated that mutational and neoantigen load (>100 non-synonymous somatic mutations) were correlated with therapeutic benefit from ipilimumab [199].

Lymphocyte infiltration in tumour biopsy samples has been associated with improved survival of patients with a range of cancers, including melanoma. The correlation between tumour-infiltrating lymphocytes and response to pembrolizumab in patients with melanoma was analysed in the KEYNOTE-001 study [200]. Pre-treatment tumour samples detected higher CD8+ (but not CD4+) T-cell densities in responding patients than in those with disease progression. Similarly, an increase in CD8+ T-cell density was also seen in serial biopsy samples of tumours during anti-PD-1 treatment in the responding group, but not in the disease progression group. However, as baseline CD8+ T-cell density may overlap between respondents and those with disease progression, the identification of an absolute cutoff as a clinically useful predictive biomarker is an unmet need.

## 7. Conclusions

The identification and the study of biomarkers in melanoma is an ever-expanding field, with promising recent findings. The use of LDH and S100 β in prognostication and monitoring of disease is well established. Other serum biomarkers, such as MIA and VEGF, have been associated with advanced stages of disease and worse prognosis, but their use is limited due to low specificity. On the other hand, DNA markers, such as *BRAF* and *NRAS*, provide well-established associations with patient selection and predict response to target therapy. ctDNA and miRNAs or lncRNAs are providing an effective insight into tumours’ genetics and helping with understanding of the pathophysiology of the disease, and hold the great advantage of allowing serial, non-invasive sampling for disease monitoring.

## Figures and Tables

**Table 1:** Serum biomarkers.

	Biomarker	Correlation	Limitation	Laboratory Methodology	References
Enzymes	LDH	Increased levels with worse prognosis Increased LDH levels with distant metastases are classed as stage IV M1C in AJCC Radiological response to immunotherapy on LDH decrease	LDH can be elevated in other conditions	Photometric assay	[4,28,30]
	Tyrosinase	May correlate with melanoma relapse	Conflicting results in studies done	RT-PCR	[15,47,48,49,50,51]
Secreted proteins/antigens	MIA	Increased levels with advanced disease and worse prognosis	Low specificity in newly diagnosed metastatic melanoma	ELISA	[5,6,40]
	TA90 Antigen	May be an independent predictor for survival, prognosis and recurrence	Can be elevated in inflammatory processes		[15,46]
	VEGF	Elevated in advanced stage melanoma Associated with negative immune effects Could be an independent prognostic marker for survival	Levels can also be elevated in healthy individuals Low sensitivity, specificity and positive predictive value in monitoring	ELISA, RT-PCR	[41,42,44,45]
	Osteopontin	May be used in conjunction with S100b to predict relapse of high risk for metastases	Can be found in autoimmune conditions	IHC, TMA	[52,53,54,55]
	IL-8	Increased levels with disease stage, survival tumour burden and response to treatment	Can be elevated in other inflammatory processes	ELISA, IHC, RT-PCR, TMA, HPLC	[56]
	MAGE-A3	Elevated levels can be found in early melanoma stages	May be elevated in other tumours Its use as a prognostic factor is not yet proven	RT-PCR	[57,58]
	Glycoprotein YKL-40	Found to be an independent prognostic marker correlating with disease-free and overall survival	Has not received FDA authorisation Can yield false-negative results Can be associated with other tumours or inflammatory processes	ELISA	[59,60,61,62,63]
	CYT-MAA	Linked with disease recurrence and progression May be related to response to immunotherapy	Not sensitive or specific		[64]
	MTF	Thought to contribute to angiogenesis, tumour proliferation	It is also excreted in exocrine tissues	ELISA, IHC, RT-PCR	[64,65,66,67]
	MITF	Has a diagnostic role in melanoma Increased levels with reduced invasiveness	Exact physiological role not yet discovered	ELISA, IHC, RT-PCR, HPLC	[68,69,71]
	GP100	Increased levels are found in neonatal cells and melanoma cells	Not specific Not proven to correlate with response to treatment	ELISA, IHC, RT-PCR	[72,73,74]
	CRP	Elevated CRP and IL6 are linked to reduced survival and treatment resistance CRP may be an independent predictor for survival	Can also be elevated by a multitude of other factors	IP	[74,75,76]
S100 Proteins	S100 β	Increased levels with reduced survival May be related with disseminated disease Recommended in some German and Swiss guidelines as surveillance	S100b can also be elevated in liver, brain, renal injury, inflammatory and infectious conditions	ELISA, LIA	[32,33,35,36,37]

AJCC: American Joint Committee on Cancer; RT-PCR: reverse transcription polymerase chain reaction; ELISA: enzyme-linked immunosorbent assay; IHC: immunohistochemistry; TMA: tissue microarray; IL-8: interleukin-8; HPLC: high performance liquid chromatography; MAGE-A3: MAGE Family Member A3; CYT-MAA: cytoplasmic melanoma-associated antigen; MTF: melanotransferrin; MITF: microphthalmia-associated transcription factor; GP100: glycoprotein 100; CRP: C reactive protein; IP: immunoprecipitation; LIA: luminescence immunoassay.

**Table 2:** DNA Markers and Molecular liquid biomarkers.

Biomarker	Correlation	Limitation	Laboratory Methodology	References
*BRAF* mutations	superficial spreading subtype, younger patient age, and skin sites without chronic sun-induced damage shorter overall survival in patients with stage IV disease and early disease BRAF inhibitors have promising results	Resistance to BRAF inhibitors	Droplet digital PCR, AS-PCR or ARMS, BEAMing technology	[8,92,95,99,102,105]
ctDNA	ctDNA correlates with disease stage In coexistence with *BRAF/NRAS*, mutations may predict treatment resistance LOH of ctDNA correlates with worse prognosis Easier sampling than tumour specific markers	ctDNA levels vary and are influenced by many variables	Droplet digital PCR, NGS	[10,174,176,177,181]
*CDKN2A* mutations	The most common alteration in hereditary melanoma Associated with more aggressive spreading	Survival rates are not replicated in different studies	Droplet digital PCR, NGS	[83,86,96,158]
*NRAS* mutations	Typically related to UV exposure in older individuals Mutations may be found in melanocytic and dysplastic naevi Associated with higher tumour stages	Unclear prognostic value Effective NRAS inhibitors not yet developed	Droplet digital PCR, BEAMing technology	[96,104,112,117,118,120]
*BAP1* mutations	Often seen in metastatic uveal melanoma Associated with worse prognosis and risk of metastasis *BAP1*-tumour predisposition syndrome	No therapeutic targeting available yet *BAP1* role not fully understood yet	Droplet digital PCR, NGS	[96,104,132,162,163]
*KIT* mutations	Mediates cell growh, proliferation, invasion, cell survival Found in mucosal, skin and acral melanomas Associated with worse survival	Rare mutations (1–7%) Awaiting trials with Imatinib	Droplet digital PCR, AS-PCR or ARMS, BEAMing technology	[96,114,150]
*GNAQ/GNA11* mutations	Can be useful in diagnosing uveal melanoma/ differentiating May be related to shorter survival	Are rarely found in cutaneous melanoma Use as a prognostic marker not established	Bi-PAP	[96,134,135,136,137]
*NF-1* mutations	Can be found in desmoplastic and cutaneous melanomas co-existence of BRAF mutation may increase resistance	Range of mutation presence in DM varies from 45–93%	Droplet digital PCR, NGS	[99,140,141,142,143]
PI3K/AKT/mTOR Pathway mutations	Regulate critical processes relating to cellular growth and survival	mTOR inhibitors have not demonstrated significant benefit	Droplet digital PCR, NGS	[146,147,149]
miRNAs and lncRNAs	Diagnostic, prognostic and predictive value in melanoma Elevated levels of miRNA-221 have been observed in early melanomas increasing miRNA-221 levels further correlated with increased stage lncRNAs are upregulated in melanoma compared to normal controls, and significantly higher at later stage (stage III and IV) compared to early-stage melanomas (stage I and II)	Not tumor specific and it is difficult to attribute whether changes in abundance are due to the cancer or to secondary conditions such as inflammation	Droplet digital PCR	[187,188,189,190,191,192,193,194,195,196]

ctDNA: circulating tumor DNA; miRNAs: microRNAs; lncRNAs: long noncoding RNAs; UV: ultraviolet; LOH: loss of heterozygosity; PCR: Polymerase chain reaction; AS-PCR: allele-specific PCR; ARMS: allele-specific amplification refractory mutation system PCR; BEAMing: bead emulsification amplification and magnetics; NGS: next generation sequencing; Bi-PAP: mutation-specific bidirectional pyrophosphorolysis-activated polymerization.
